# Improving intelligent dasymetric mapping population density estimates at 30 m resolution for the conterminous United States by excluding uninhabited areas

**DOI:** 10.5194/essd-14-2833-2022

**Published:** 2022-06-23

**Authors:** Jeremy Baynes, Anne Neale, Torrin Hultgren

**Affiliations:** 1 Center for Public Health and Environmental Assessment, US Environmental Protection Agency, Research Triangle Park, NC 27711, USA; 2 EPA National Geospatial Support Team, ITS-EPA III Infrastructure Support and Application Hosting Contract, Research Triangle Park, NC 27711, USA

## Abstract

Population change impacts almost every aspect of global change from land use, to greenhouse gas emissions, to biodiversity conservation, to the spread of disease. Data on spatial patterns of population density help us understand patterns and drivers of human settlement and can help us quantify the exposure we face to natural disasters, pollution, and infectious disease. Human populations are typically recorded by national or regional units that can vary in shape and size. Using these irregularly sized units and ancillary data related to population dynamics, we can produce high-resolution gridded estimates of population density through intelligent dasymetric mapping (IDM). The gridded population density provides a more detailed estimate of how the population is distributed within larger units. Furthermore, we can refine our estimates of population density by specifying uninhabited areas which have impacts on the analysis of population density such as our estimates of human exposure. In this study, we used various geospatial datasets to expand the existing specification of uninhabited areas within the United States (US) Environmental Protection Agency’s (EPA) EnviroAtlas Dasymetric Population Map for the conterminous United States (CONUS). When compared to the existing definition of uninhabited areas for the EnviroAtlas dasymetric population map, we found that IDM’s population estimates for the US Census Bureau blocks improved across all states in the CONUS. We found that IDM performed better in states with larger urban areas than in states that are sparsely populated. We also updated the existing EnviroAtlas Intelligent Dasymetric Mapping toolbox and expanded its capabilities to accept uninhabited areas. The updated 30 m population density for the CONUS is available via the EPA’s Environmental Dataset Gateway (Baynes et al., 2021, https://doi.org/10.23719/1522948) and the EPA’s EnviroAtlas https://www.epa.gov/enviroatlas, last access: 15 June 2022; Pickard et al., 2015).

## 1 Introduction

Population density is a critical variable for understanding human–environment relationships. It has been recognized as an essential societal variable for studying human interactions with the environment, and it is crucial for quantifying human exposure to natural hazards. Data on population density have facilitated global mapping of the changing human footprint on Earth’s terrestrial surface (Venter et al., 2016). The drivers and patterns of human settlement and population growth are a key part of understanding this expanding human footprint. Population density data allow researchers to investigate the spatiotemporal patterns of human settlement, monitor changes in those patterns, and investigate how urban areas expand (Fang et al., 2018; Wei et al., 2017; Fang and Jawitz, 2019; Taubenböck et al., 2019). Furthermore, population density maps have allowed researchers to identify natural drivers of population density such as elevation, temperature, and precipitation (Liu et al., 2019; Samson et al., 2011). Population density data offer insights into the impact of human settlement and the risks and exposure people face from the environment. Population density has been used to assess the impacts of human activity on coral reefs (Bellwood et al., 2012; Cinner et al., 2013; Morais et al., 2019). Considerable work has used population density data to quantify human exposure and vulnerability to natural disasters and pollution (Smith et al., 2019; Nicholls and Small, 2002; Carroll et al., 1997; Samoli et al., 2019; Nahayo et al., 2019; Nasiri et al., 2018; Yuan et al., 2019). For example, population data have been used to quantify US population exposure to fine particles as part of reporting the costs and benefits of the Clean Air Act Amendments of 1990 (US Environmental Protection Agency, 2011). In Vietnam, researchers identified critical values of population density where the risk of dengue fever is high (Schmidt et al., 2011). Globally, population density was found to be a significant driver of the origins of emerging infectious diseases from 1940–2004 (Jones et al., 2008).

In the United States (US), estimating population density usually involves distributing population counts collected within source units such as blocks, or block groups delineated by the US Census Bureau. The Census Bureau, like many other organizations, relies on censuses and surveys to allocate people to source units. Population density is often simply estimated as the population count divided by the area for each source unit. However, the population recorded in these units can be disaggregated to provide estimates of how the population within source units is distributed. This disaggregation is important when source units are large, varying in shapes and sizes, or the population within the source units is not evenly distributed (Leyk et al., 2019). Various techniques have been used to allocate population counts from source units to estimate population density. Pycnophylactic interpolation estimates population density within source units using a grid of equally sized cells (Tobler, 1979). The pycnophylactic property of this method ensures that the counts from each source unit are maintained in the process and that population is not lost or displaced beyond the source unit within which it was recorded (Tobler, 1979). Source units can be divided up into smaller target units of homogenous population density. For example, target units can be determined by the spatial intersection between census blocks and land cover classes. In this example, a target unit consists of the area of a land cover class inside a census block. Areal weighting distributes the population of source units to target units by the proportion of the area of the target unit inside the source unit (Goodchild and Lam, 1980). This method maintains the counts of the source units as suggested by Tobler (1979). However, the only determinant of population density is the area of a target unit inside a source unit. This is problematic where area might not be the best indicator of population dynamics. For example, in a source unit that is largely covered by a wildlife refuge and minimally covered by urban land use, the proportion of the source unit’s population that resides in urban land use should, in reality, be greater than that in the wildlife refuge.

Dasymetric allocation of population can incorporate the population dynamics that are to be expected within source units in order to estimate population density. Dobson et al. (2000) used coefficients calculated by weighted combinations of factors that influence human populations to estimate population density from aggregate population counts. Other methods have used the random forest algorithm to predict population density at fine scales using aggregate population counts and aggregated fine-scale covariates that are related to population density (Sorichetta et al., 2015; Stevens et al., 2015). Researchers have modeled gridded population density from small-area sampling of population counts rather than using a national census (Weber et al., 2018). To improve estimates, various dasymetric population mapping methods have used land use–land cover; climatic and topographic variables such as temperature, precipitation, elevation, and slope; and socio-economic variables such as nighttime lights, roads, and points of interest related to human activity (Karunarathne and Lee, 2019; Lloyd et al., 2019; Ye et al., 2019). Dmowska and Stepinski (2017) used dasymetric modeling with a hybrid land cover and land use map to produce a US-wide grid of population density at 30 m resolution. Their effort estimated land cover densities using nationwide sampling of homogeneous census blocks but left open the possibility that local sampling from smaller spatial extents could improve results.

Mennis and Hultgren (2006) developed an intelligent dasymetric mapping (IDM) technique that estimates population density by determining class-specific representative population densities from an ancillary raster containing classes that are indicative of population dynamics. IDM relies on a limited number of required input datasets, an ancillary raster, and population source units. This makes IDM an appealing method over other promising, but more complex, methods (e.g., machine learning) because of its usability among broad audiences and applicability at various locations and scales. In 2016, IDM was used to develop a dasymetric population map of the conterminous US (CONUS) by the Environmental Protection Agency’s (EPA) Office of Research and Development. The map was developed for EnviroAtlas, an online collection of interactive tools and resources that provides data, research, and analysis on the relationships between nature, people, health, and the economy (Pickard et al., 2015). Census block counts for 2010 were disaggregated to 30 m grid cells using the 2011 National Land Cover Database (NLCD) as the ancillary raster. The identification of uninhabited areas and not allocating people to those areas can further refine population density to areas where humans are more likely to settle. This refinement has a marked impact on the accuracy of estimates of population density (Fang and Jawitz, 2018; Smith et al., 2019; Leyk et al., 2019).

Uninhabited areas in the 2016 EnviroAtlas dasymetric population map effort were identified as the open water, perennial ice/snow, and emergent herbaceous wetlands land cover classes along with areas that have a slope greater than 25%. In this study, we updated the pre-existing EnviroAtlas dasymetric population map for the CONUS by incorporating additional geospatial datasets to expand areas identified as uninhabited. We then conducted an assessment to test the validity of our methods and measure any improvement in population density mapping associated with our effort. While updating the EnviroAtlas dasymetric population map, we also updated the EnviroAtlas IDM toolbox, a toolbox originally developed for ESRI ArcMap 10.3 that allows users to create dasymetric population maps of their own study areas. The updated methodology has been implemented as a toolbox for ArcGIS Pro and a standalone Python tool that relies on open source libraries (US Environmental Protection Agency, 2022a, 2022b). We expanded the IDM toolbox’s capabilities to accept additional uninhabited areas from users.

## 2 Data

We updated the existing population density map for CONUS using data that were nationally consistent and complete, fit for purpose, freely available or available under existing license, and relevant to human land use. Table 1 presents the datasets and layers that were used to update the dasymetric population map.

### 2.1 Boundaries

The TIGER/Line shapefiles from the United States Census Bureau provided state boundaries along with their Federal Information Processing Series (FIPS) codes (US Census Bureau, 2012). The boundaries for statistical entities from the US Census Bureau are organized hierarchically from census blocks within block groups which are contained within census tracts within the counties of a state (US Census Bureau, 2012). We used a special release shapefile of the 2010 TIGER/Line census blocks that included the population and housing counts from the 2010 decennial census carried out by the US Census Bureau (US Census Bureau, 2012). The shapefile also includes the state FIPS code, county FIPS code, the census tract code, and the census tabulation block number for each block (US Census Bureau, 2012).

### 2.2 Land cover

The 30 m, 2011 land cover classification from the 2016 NLCD (i.e., NLCD2016 2011) was used as the ancillary raster (Yang et al., 2018; Homer et al., 2020). Yang et al. (2018) used a leaf-on Landsat image as the base image for the 2011 NLCD classification. Pixels with cloud, shade, and other anomalies in the base Landsat image were filled using leaf-on or leaf-off Landsat images within 2 years of the base image (Yang et al., 2018). The NLCD classification was carried out using a decision-tree classifier with the Landsat image and ancillary data (Yang et al., 2018). The overall user accuracy for NLCD2016 2011 is 86.8% (Wickham et al., 2021)

### 2.3 Land use

In order to identify uninhabited areas, we used several publicly available and proprietary datasets from the OpenStreetMap Foundation & Contributors (OSM), NAVSTREETS, CoreLogic, the Protected Areas Database of the US (PAD-US), the North American Rail Network (NARN), NLCD, and the National Elevation Dataset (NED; OpenStreetMap contributors, 2019; CoreLogic, 2018; HERE, 2017; Yang et al., 2018; US Geological Survey, 1999). From these data, we used several vector features and rasters related to built structures, zoning, topography, and protected areas. Volunteers contribute and maintain geospatial data about roads, rail roads, built structures, land use, parks, and various other categories for OSM (OpenStreetMap contributors, 2019). NAVSTREETS provides boundaries for built structures and land use, and Core-Logic provides boundaries for residential and non-residential parcels (CoreLogic, 2018; HERE, 2017). PAD-US is produced by the United States Geological Survey (USGS) Gap Analysis Program and provides nation-wide spatial data outlining the boundaries of protected open space held by national, state, and regional/local governments and non-profit conservation organizations (US Geological Survey, 2018; Gergely and McKerrow, 2016). NARN is managed by the Federal Railroad Administration and is a comprehensive database of the US railway system (Federal Railroad Administration, 2019). NLCD includes a developed impervious descriptor product that classifies the NLCD’s percent impervious product into types of roads and energy production (Yang et al., 2018). The impervious product was developed by MRLC using regression tree models with Landsat imagery and training datasets generated from nighttime light imagery (Yang et al., 2018).

## 3 Methods

### 3.1 Uninhabited features

Uninhabited features were identified and prepared for each CONUS state and Washington, DC. The goal of this step was to produce a single layer of uninhabited features for each state that would be used to reclassify NLCD pixels to a new uninhabited land cover class. From NAVSTREETS, we identified shopping centers, industrial complexes, cemeteries, aircraft roads, and rail roads as uninhabited. A 30 m buffer was created around aircraft road centerlines, and a 15 m buffer was created around railroad centerlines to ensure that all line features were converted to raster. Because we could find no existing rail yard polygon data, rail yard polygons were derived from railroad lines in NARN. We approximated rail yard extents by applying a 500 m buffer around all rail line features with “YARDS” in the name field and then dissolved the resulting polygons into one feature. We then applied a negative 480 m buffer to the results of the 500 m buffer to ensure we were not capturing areas outside the extent of the rail lines. These areas were identified as uninhabited. From OSM we identified retail, commercial land use, malls, industrial complexes, supermarkets, and schools as uninhabited (Table 1). Additionally, we designated local parks, state parks, state forests, national wildlife refuges, national forests, national parks, national lakeshore or seashore, and national grasslands from PAD-US as uninhabited (Table 1).

The possibility of housing within the areas we identified as uninhabited warranted additional attention before marking the entire area as uninhabited. For example, national forests have experienced an estimated housing growth of about 940 000 units between 1940 and 2000 within their boundaries (Radeloff et al., 2010). In order to allocate potential population within areas identified as uninhabited, we removed (i.e., spatially clipped) areas covered by residential parcels within all uninhabited features listed in Table 1. We used the residential parcels from the area parcel feature class from CoreLogic (2018). Residential parcels in this dataset included typical single-family residences; however, multi-family dwellings including apartment complexes, urban mixed use, and retirement communities were often considered commercial properties. We found no consistent method to isolate these multi-family-inhabited land-use parcels from other uninhabited commercial parcels; therefore, we could not identify all commercial parcels as uninhabited.

Mixed-use zones may contain census blocks with a mix of retail, commercial, civic, business, industrial, and residential land uses (Moos et al., 2018; Song and Knaap, 2004). Several of the land use types we identified as uninhabited can exist in mixed-use zoning and thus potentially be inhabited. From OSM and NAVSTREETS, we labeled shopping centers, industrial complexes, malls, and supermarkets along with retail and commercial land uses as areas we initially identified as uninhabited that can be found in mixed-use zoning (Table 1). If the combined area of these features covered greater than 90% of the entire census block area, that block was labeled as mixed use, and those features within that block were excluded from our uninhabited features.

**Figure 1.**
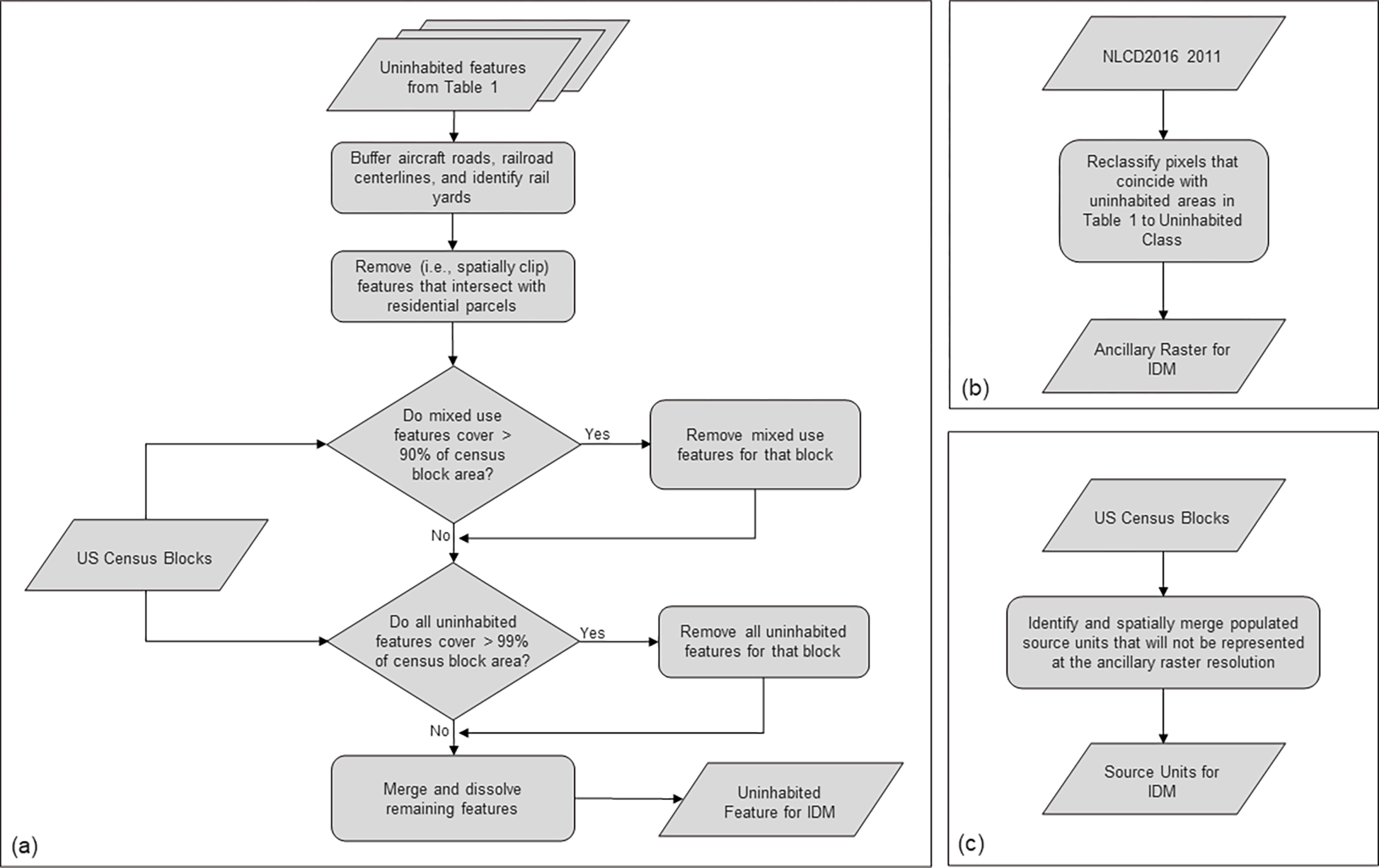
Data preparation workflow for uninhabited features **(a),** ancillary raster **(b)**, and source units **(c)** for IDM processing.

Furthermore, if the combined area of features we identified as uninhabited covered more than 99% of a census block, all features within that block were excluded from our uninhabited features. This way, if a census block was covered almost entirely by uninhabited features, any population recorded in that block would not be lost. Uninhabited vector features remaining after excluding residential parcels, mixed-use features, and features that covered more than 99% of a block were projected onto an Albers equal-area conical projection and used as the uninhabited features for IDM (Fig. 1a). The updated IDM toolbox reclassifies ancillary raster pixels that coincide with uninhabited features to a new uninhabited ancillary class.

### 3.2 Ancillary raster

NLCD2016 2011 was the basis for the ancillary raster. We retained the only non-land cover attribute for identifying uninhabited areas from the 2016 EnviroAtlas dasymetric population map; areas with a slope of greater than 25% were considered uninhabited. The percent slope was calculated from the National Elevation Dataset using GDAL (GDAL/OGR contributors, 2019). In addition to slope, we used other gridded datasets to mask uninhabited areas. Land cover pixels that coincided with uninhabited area pixels from Table 1 (i.e., primary roads and energy production classes from the developed imperviousness descriptor, or a slope of greater than 25 %) were reclassified to a new uninhabited land cover class. This reclassified NLCD classification was used as the ancillary raster for IDM (Fig. 1b).

### 3.3 Source units

The US Census Bureau blocks with associated population counts from the 2010 decennial census were used as source units for IDM. The IDM toolbox converts source units into a raster that matches the spatial resolution and extent of the input ancillary dataset. Small or irregularly shaped source units that do not coincide with the center of a pixel at the ancillary dataset resolution will not be represented in the derived raster, and the population in that unit will not be included in the estimate of population density. To account for the population in these blocks, we identified any populated census block that would not be represented in a 30 m × 30 m pixel. These blocks were spatially merged and had their population added to the neighboring block that met all the following criteria:

1. had the longest shared border,
2. was in the same census tract,
3. had a population greater than zero.

If no neighboring block in the same census tract had population, then criteria 3 was dropped. This allowed us to account for population in these small blocks while not displacing the population outside of the census tract and limiting displacing population into unpopulated blocks. Census blocks were projected onto an Albers equal-area conical projection to match the NLCD. This modification of the 2010 census blocks was used as the source units for IDM (Fig. 1c).

### 3.4 Intelligent dasymetric mapping

The IDM method from Mennis and Hultgren (2006) was used to estimate the population density (people per pixel). The modified 2010 US Census Bureau blocks with associated population were used as source units, and the NLCD reclassified to incorporate uninhabited areas was used as the ancillary raster. The target units were created by the spatial intersection between NLCD classes and US Census Bureau blocks. Therefore, each target unit consists of the area of an NLCD class inside a block. A homogenous gridded population density (30 m × 30 m) was estimated for each target unit inside the census blocks.

In order to estimate the population density for the target units, a representative population density was estimated for each land cover class from NLCD for each state. The representative population density of a land cover class is the number of people per pixel that were expected to reside in that land cover class throughout the state. IDM offers three ways to estimate the representative population density for an ancillary class. First, a representative population density can be set for an ancillary class from expert or domain knowledge or previous research. In line with the 2016 specification of uninhabited areas, the representative population density for the following land cover classes from NLCD was preset to zero people per pixel: open water, perennial ice/snow, and emergent herbaceous wetlands. Since we added an additional “uninhabited” class to the NLCD classification, we also set the preset density for this class to zero people per pixel. Second, the representative population density for an ancillary class can be sampled from source units that are considered representative of that ancillary class. The IDM toolboxes we developed allow users to set sampling eligibility thresholds. For this effort we determined that a representative block, *b*, for a sampled land cover class, *s*, met the following criteria.

1. A total of 95% of the area of the source unit *b* was covered by land cover class *s*.
2. The area of source unit *b* was greater than 900m^2^ (1 pixel).

At least three representative census blocks were required for a land cover class to be considered sampled. After collecting all the representative blocks for a sampled land cover class, the representative population density for the class was estimated as (Mennis and Hultgren, 2006)

(1) $$\hat{D_{\text{s}}}=\sum_{\text{b}=1}^{m}y_{\text{b}}/\sum_{\text{b}=1}^{m}A_{\text{b}},$$

Where $\hat{D_{i}}$ is the representative population density of sampled land cover class *s*, *y*_b_ is the population count of census block *b*, *A*_b_ is the area of census block *b*, and *m* is the number of representative blocks for class *s*.

Since the entire area of the block is used to distribute population counts in Eq. (1), only using blocks where 95% of the area is covered by the sampled land cover class ensures that the representative population density estimated for the class is based on homogenous blocks. Lastly, intelligent areal weighting (IAW) was used to calculate the representative population density for all land cover classes within each state where insufficient representative blocks were found and no representative population density was preset. By this point, a representative population density had been determined by either a sampled or preset representative population density for land cover class *k* (i.e., {*k* ∈ *C*|*k* ∈ (*P* ∪ *S*)}, where *C* is the set of all ancillary classes, *P* is the set of all preset ancillary classes, and *S* is the set of all sampled ancillary classes). IAW calculates the remaining population counts for each source unit after sampled and preset representative population densities have determined a population estimate for target units in the source unit when possible (Mennis and Hultgren, 2006):

(2) $$G_{\text{b}}=y_{\text{b}}-\sum_{\text{t}(k)\in b}\hat{D_{k}}A_{\text{t}(k)},$$

where *G*_b_ is the remaining census population count for block *b*, $\hat{D_{k}}$ is the representative population density of land cover class *k*, and *A*_t(*k*)_ is the area of the target unit associated with land cover class *k* in census block *b*.

After calculating the remaining population for each block, an initial population was allocated to a given block’s target units associated with land cover class *i* that had not been determined by either a sampled or preset representative population density (i.e., {*i* ∈ *C*|*i* ∉ (*P* ∪ *S*)}). IAW uses areal weighting to distribute the remaining census counts to the remaining target units (Mennis and Hultgren, 2006):

(3) $${\hat{y}}_{\text{t}(i)}=\{\begin{array}{r} 0,\;\text{if}\;G_{\text{b}}<0\\ G_{\text{b}}\left(A_{\text{t}(i)}/\sum_{\text{t}(i)\in \text{b}}A_{\text{t}(i)}\right),\;\text{if}\;G_{\text{b}}\geq 0 \end{array},$$

where ${\hat{y}}_{\text{t}(i)}$ is the initial estimated population count for the target unit associated with land cover class *i* in block *b*, and *A*_t(*i*)_ is the area of the target unit associated with land cover class *i* in block *b*.

Equation (3) differs slightly from the methods of Mennis and Hultgren in that here an initial population of zero was allocated to unsampled land cover classes if the total population estimated for sampled or preset classes in the block exceeded the census count for the block. Although not explicitly stated in Mennis and Hultgren, this was implied as it avoids negative population estimates attributed to target units. After the initial population counts were estimated for each target unit associated with land cover class *i*, the representative population density of land cover class *i* was determined as (Mennis and Hultgren, 2006)

(4) $$\hat{D_{i}}=\sum_{\text{t}(i)=1}^{p}{\hat{y}}_{\text{t}(i)}/\sum_{\text{t}(i)=1}^{p}A_{\text{t}(i)},$$

where $\hat{D_{i}}$ is the representative population density of land cover class *i*, and *p* is the number of target units in the study area that are associated with land cover class *i*.

After the representative population density for each land cover class was determined using either a preset density, sampling (Eq. 1), or IAW (Eq. 4), the final population estimate for target unit *t*, which consists of the area of a land cover class *c* (i.e., {*c* ∈ *C*}) inside block *b*, was calculated as (Mennis and Hultgren, 2006)

(5) $${\hat{y}}_{\text{t}}=\{\begin{array}{c} y_{\text{b}}\left(A_{\text{t}}/\sum_{\text{t}=1}^{\text{n}}A_{\text{t}}\right),\;\text{if}\;\sum_{\text{t}=1}^{\text{n}}\hat{D_{\text{c}(\text{t})}}=0\\ y_{\text{b}}\left(A_{\text{t}}\hat{D_{\text{c}(\text{t})}}/\sum_{\text{t}=1}^{\text{n}}A_{\text{t}}\hat{D_{\text{c}(\text{t})}}\right),\;\text{if}\;\sum_{\text{t}=1}^{\text{n}}\hat{D_{\text{c}(\text{t})}}>0 \end{array},$$

where ${\hat{y}}_{\text{t}}$ is the population estimated for target unit *t* associated with land cover class *c* in block *b*, *n* is the number of target units in block *b*, *A*_t_ is the area of target unit *t*, and $\hat{D_{\text{c}(t)}}$ is the representative population density of land cover class *c* associated with target unit *t*.

Equation (5) ensured that the population was not displaced beyond the block (Mennis and Hultgren, 2006). Equation (5) is also a slight deviation from Mennis and Hultgren in that area weighting would be used for population within a block made up entirely of land cover classes with representative population densities estimated at or preset to zero. Although rare, there were instances of populated census blocks composed entirely of these land cover classes. This modification ensured any population within these blocks was not lost without giving weight to any specific land cover class. The final population density for a target unit *t* that is associated with ancillary class *c* and source unit *b* can be calculated as (Mennis and Hultgren, 2006)

(6) $${\hat{d}}_{\text{t}}={\hat{y}}_{\text{t}}/A_{\text{t}},$$

where ${\hat{d}}_{\text{t}}$ is the population density (people per pixel) estimated for target unit *t*.

**Figure 2.**
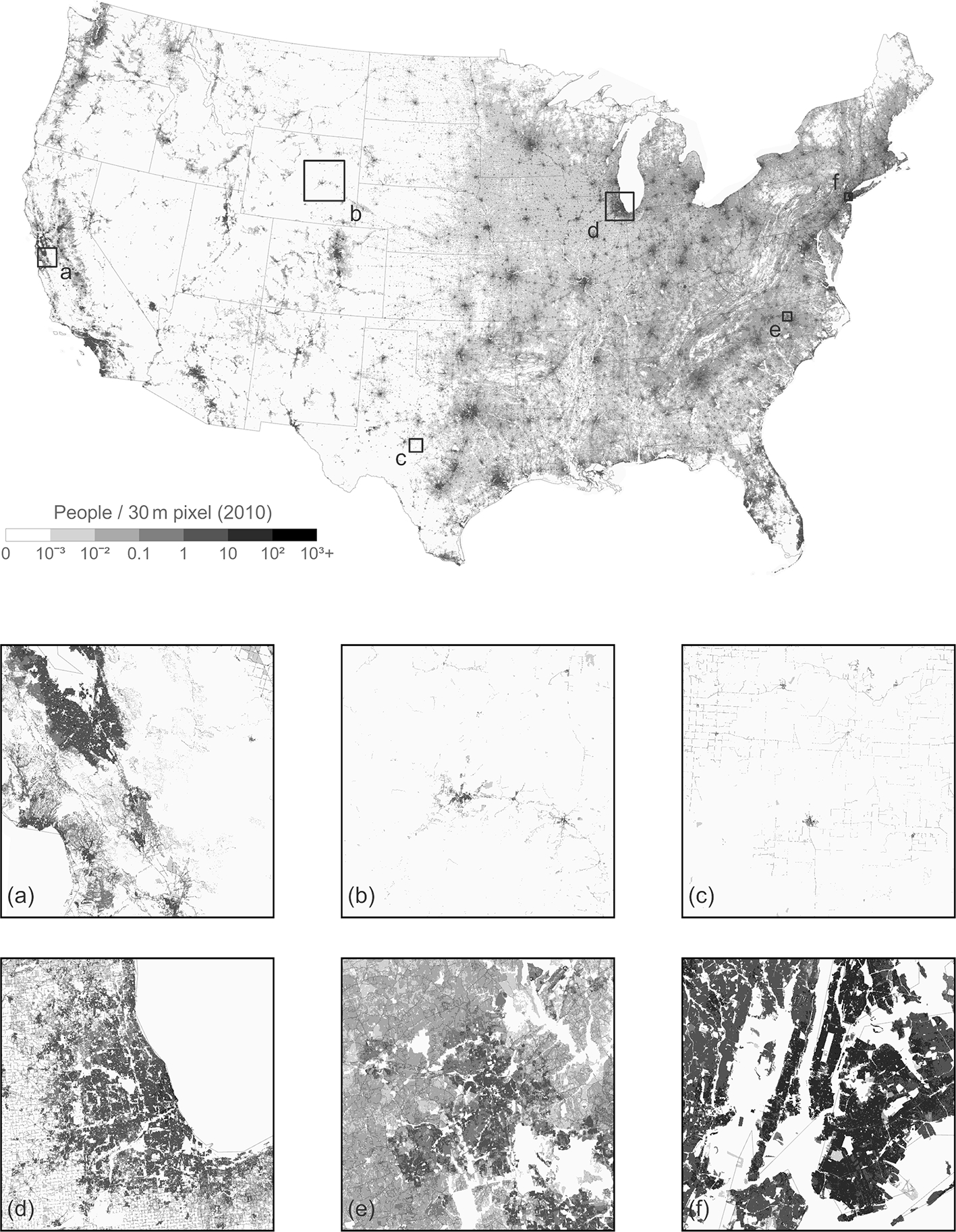
Population density estimated by intelligent dasymetric mapping at 30 m spatial resolution for the conterminous United States and areas around **(a)** Santa Clara County, CA; **(b)** Natrona and Converse counties, WY; **(c)** Concho County, TX; **(d)** metropolitan Chicago, IL; **(e)** Durham County, NC; and **(f)** metropolitan New York City, NY.

We chose to apply IDM using sub-national zones versus a national analysis. States were selected as zones because they are generally large enough to collect a suitable number of homogenous source units for sampling while being small enough to represent some of the heterogeneity in population density across the CONUS. The input blocks, uninhabited features, and land cover rasters were prepared for each CONUS state and Washington, DC. In order to increase the number of representative blocks, all data for Rhode Island were combined with neighboring Massachusetts. Likewise, data for Washington, DC, were combined with Maryland. Representative population densities were determined for 17 land cover classes in 47 “states” in the US for a total of 799 estimated densities (Table 2). Four land cover types were preset at zero for every state. Of the 611 unique land cover type–state combinations that were not initially preset at zero, 596 were determined with sampling, 14 were determined using IAW, and one was preset (Table 2). In Connecticut, the representative population density for scrub/shrub was estimated at 3.4 using IAW. This would have resulted in shrub/scrub having the highest representative population density for any land cover type in the state, and the estimate was over 6 standard deviations above the mean for that land cover type in all states. We chose to rerun IDM for Connecticut using the average representative density for scrub/shrub from all other states as a preset density. Population density was determined for each NLCD pixel within each state and then joined to create a seamless 30 m population density estimate for the CONUS (Fig. 2).

### 3.5 Assessment

**Figure 3.**
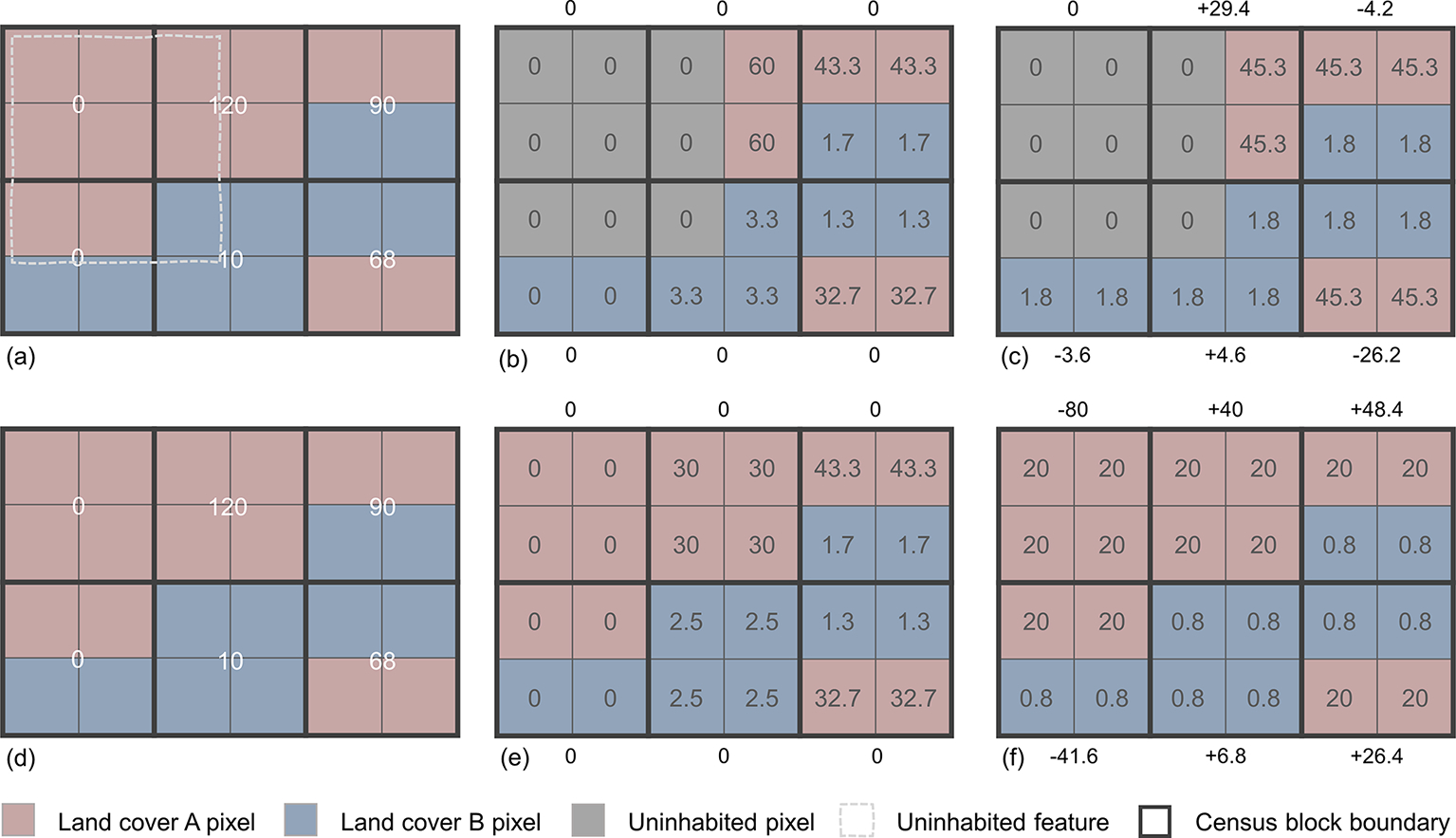
A simulated illustration of six census blocks and associated population within a single census tract **(a, d)**. This tract has two land cover types, A and B, with representative population densities estimated at 5.0 and 0.2, respectively, and an uninhabited feature that is new to the updated specification of uninhabited areas. Block level errors are provided adjacent to each block. Because our method has no cumulative error at the block level **(b, e)**, we assessed our representative population densities by applying the densities at the tract level (i.e., no cumulative error at the tract level) with the updated specification of uninhabited areas **(c)** and the 2016 specification of uninhabited areas **(f)**. In this illustration the tract has a MAE of 40.6 with the 2016 specification of uninhabited areas **(f)** and 11.3 with updated specification of uninhabited areas **(c)**. Note that for illustrative purposes in this figure, we used the same representative population density estimates for both updated **(a–c)** and 2016 **(d–f)** specifications. In practice the representative population density estimates for the updated and 2016 specifications were determined independently and most likely would have been different.

The method we described above results in census block estimates equal to the census block numbers reported by the US Census Bureau; therefore, there is no cumulative error at the block level. To assess the validity and accuracy of our representative population density estimates, we applied IDM to a larger source unit (i.e., census tract) using densities that we determined from the smaller source unit (i.e., census block). In other words, we disaggregated the recorded population for the census tract using block-level representative population densities (Fig. 3). We concatenated the state FIPS code, the county FIPS code, and the tract code to aggregate the census blocks by tract. The census population count for each tract was calculated by summing the census population count from all the blocks inside each tract. An IDM population estimate for each block was then calculated by summing the per-pixel population densities estimated by using tracts as source units.

Mean absolute error (MAE) and root mean square error (RMSE) were calculated to assess the error between the estimated block population and the recorded block population. RMSE was normalized by the mean block population within the summary unit (i.e., state or county) to facilitate comparison between summary units (NRMSE). These metrics were calculated for each state and county in CONUS. Additionally, these metrics were calculated for the CONUS to compare model and zone performance and to facilitate comparison with other dasymetric population mapping efforts. The metrics were calculated as

(7) $${\text{MAE}}_{\text{s}}=\frac{\sum_{\text{b}=1}^{\text{n}}\left|y_{\text{b}}\;-\;{\hat{y}}_{\text{b}}\right|}{n},$$

(8) $${\mathrm{RMSE}}_{\text{s}}=\sqrt{\frac{\sum_{\text{b}=1}^{\text{n}}{\left(y_{\text{b}}\;-\;{\hat{y}}_{\text{b}}\right)}^{2}}{n}},$$

(9) $${\text{NRMSE}}_{\text{s}}=\frac{{\text{RMSE}}_{\text{s}}}{{\bar{y}}_{\text{s}}},$$

where *y*_b_ is the census population count for block *b*, ${\hat{y}}_{\text{b}}$ is the estimated population for block *b*, *s* is the unit for which census block errors are summarized. We used state, county, and the CONUS. ${\bar{y}}_{\text{s}}$ is the mean census block population count for unit *s*, and *n* is the number of blocks in unit *s*.

We compared the RMSE and MAE between the 2016 specification of uninhabited areas and our updated specification by running IDM for all CONUS states using both specifications. The 2016 specification of uninhabited areas used a preset density of zero people per pixel for land cover classes open water, perennial ice/snow, and emergent herbaceous wetlands and included areas with a slope of greater than 25 %.

## 4 Results

### 4.1 IDM performance

**Figure 4.**
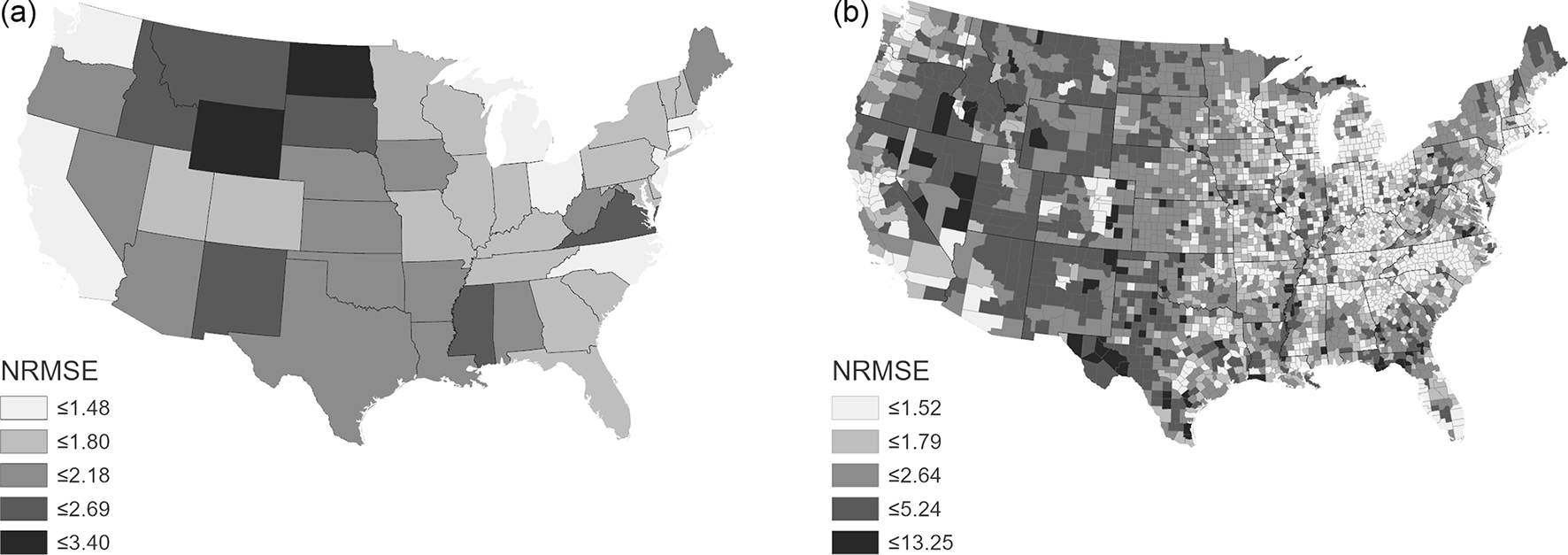
NRMSE between block population estimates and block population census counts calculated for CONUS states **(a)** and counties **(b)**. Block population was estimated by running IDM with census tracts as source units and applying representative population densities estimated by IDM using census blocks as preset densities.

**Figure 5.**
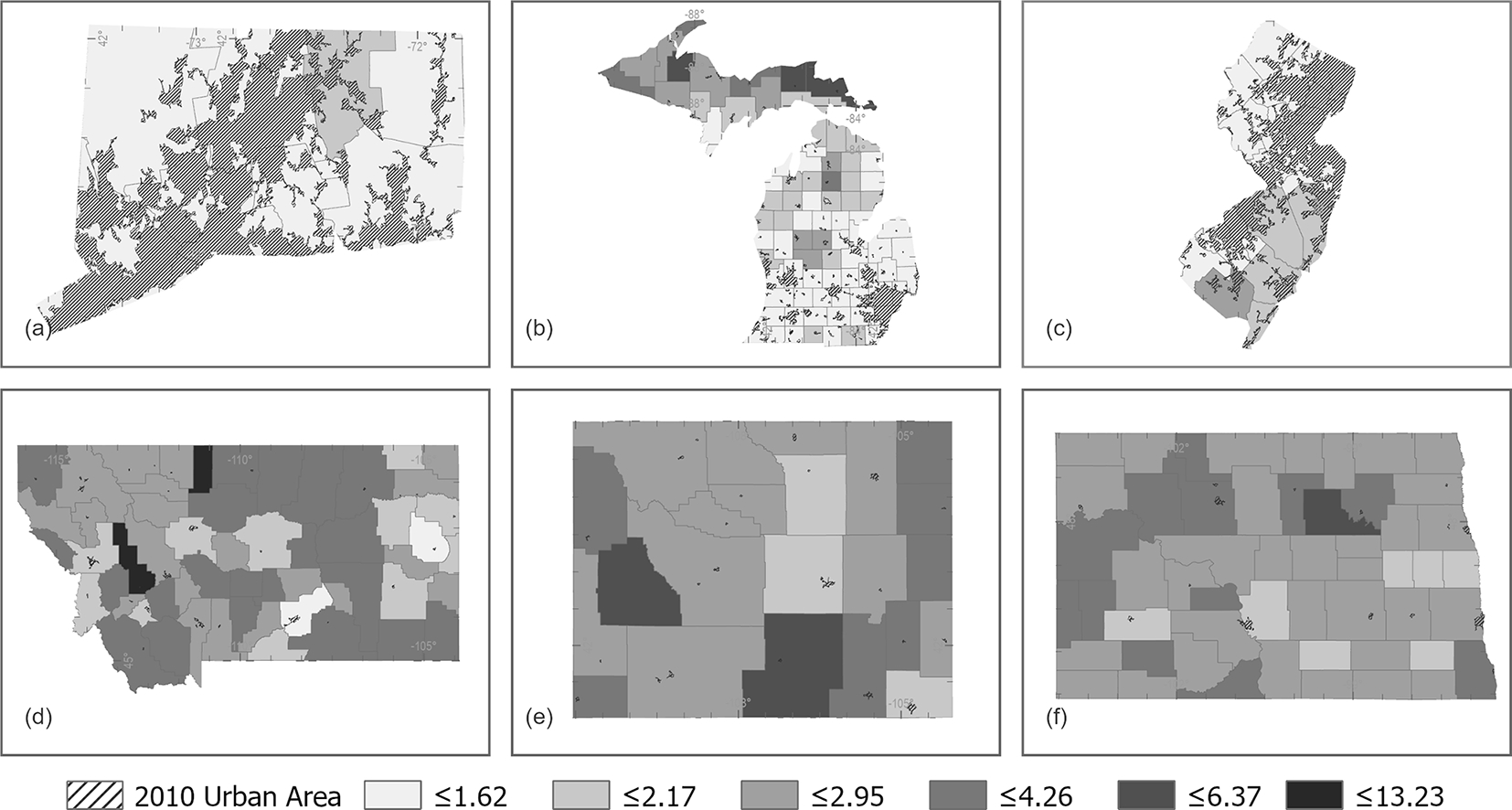
Census urban areas and county NRMSE between census block population count and the estimated block population count from IDM for some of the states with the lowest NRMSE **((a)** Connecticut, **(b)** Michigan, and **(c)** New Jersey) and highest NRMSE **((d)** Montana, **(e)** Wyoming, and **(f)** North Dakota).

NRMSE ranged from 1.21 to 3.39 (Fig. 4; Table 3). The highest state NRMSE between census block population counts and IDM estimated block population counts is for North Dakota with an RMSE that is 3.39 times the mean census block population, Wyoming with an RMSE that is 2.91 times the mean census block population, and Montana with an RMSE that is 2.60 times the mean census block population (Table 3). The lowest NRMSE between census block population counts and IDM estimated block population counts is for Connecticut with an RMSE that is 1.21 times the mean census block population, Michigan with an RMSE that is 1.36 times the mean census block population, and New Jersey with an RMSE that is 1.38 times the mean census block population (Table 3). NRMSE was summarized by state and county (Fig. 5), highlighting areas with the highest (tending towards less densely populated) and lowest values (tending towards more urban).

### 4.2 Uninhabited areas

**Figure 6.**
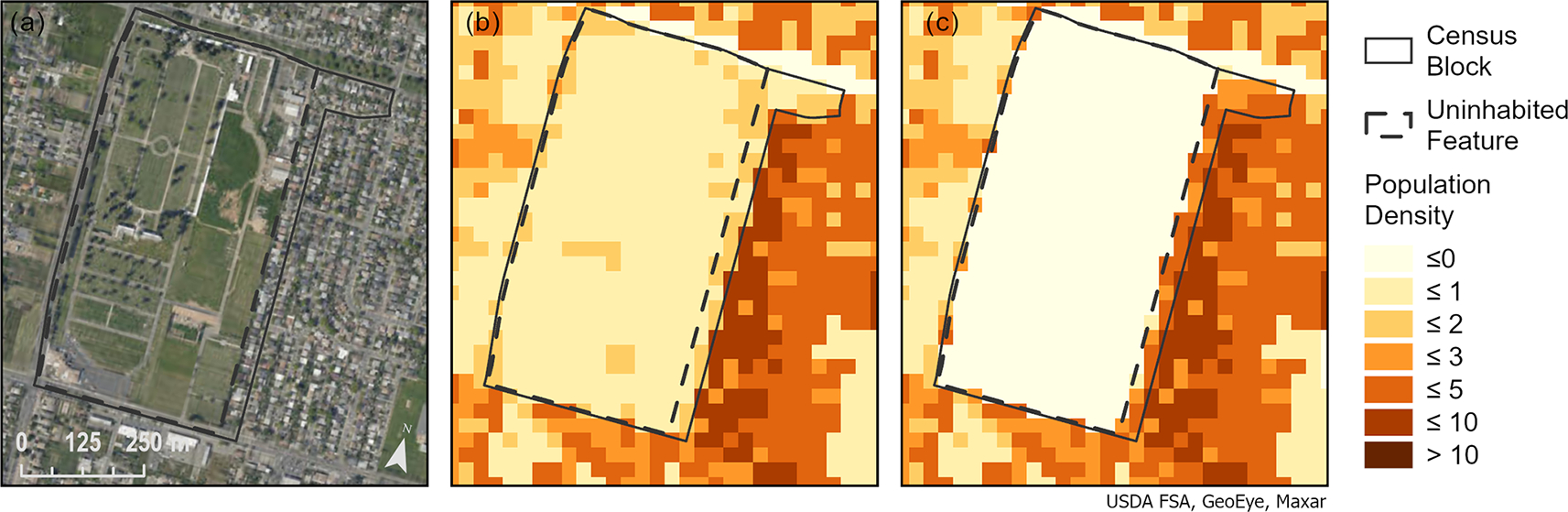
A census block near Sacramento, California, with a cemetery (i.e., uninhabited feature) covering most of the block and residential housing along the eastern border **(a).** IDM results with the 2016 specification of uninhabited **(b)** have population throughout the block while IDM results with the updated specification of uninhabited **(c)** have zero population for the cemetery and denser population along the eastern border.

The updated specification of uninhabited areas identified an additional 186 764 551 30 m pixels (~ 168000 km^2^; an area slightly less than Washington State) as having zero population in comparison to the 2016 specification of uninhabited (Table 4). Recalling that the nature of IDM does not allow for population to be displaced beyond the original source unit (i.e., census block), our updated definition reallocated approximately 9.56 million people from uninhabited areas to areas that are more likely to be inhabited (Table 4; Fig. 6).

RMSE and MAE improved for all states with the expansion of uninhabited areas (Table 3). RMSE improved by an average of 2.46 persons per census block (*σ* = 1.37), and MAE improved by an average of 1.10 persons per census block (*σ* = 0.69) across all states. The most improved states were New Jersey and New York with a difference in RMSE of −7.85 and −5.56 and a difference in MAE of −3.72 and −2.89. Some of the least improved states were North Dakota and Arkansas with a difference in RMSE of −0.76 and −0.92 and a difference in MAE of −0.23 and −0.35 (Table 3). The expanded use of uninhabited areas improved overall RMSE for the CONUS when applying IDM both with nationally determined densities and with state level densities (Table 5).

## 5 Discussion

### 5.1 IDM performance

IDM is a useful method to allocate population within heterogeneous source units. Intuitively, we would expect that identifying uninhabited areas within those source units would improve the accuracy of the allocation. Improvements in population model performance by adding variables for uninhabited areas were demonstrated by others (Fang and Jawitz, 2018). Many of the widely used models rely on multiple ancillary data layers to allocate population while acknowledging input data are often limited because of temporal constraints and necessity to cover large extents (Leyk et al., 2019). With a decrease in RMSE and MAE for every CONUS state after identifying additional uninhabited areas, we have shown that with suitable, nationally consistent data improvements in population density, estimates can be realized on regional, state, and country scales at a high spatial resolution.

Dasymetric mapping across an area as large and heterogenous as the CONUS benefited from the use of sub-national zones. Applying IDM on a state-by-state basis showed an improvement over using densities determined from a national analysis. A balance must be found in defining zones to ensure they are large enough to provide enough data to develop a useful model and small enough to maintain a suitable level of homogeneity within the zone. We attempted to further refine our product by using county-level 2013 United States Department of Agriculture Rural Urban Continuum Codes (RUCC) to create additional ancillary classes (US Department of Agriculture, 2020). RUCC has nine classes but can be collapsed to official Office of Management and Budget metro and nonmetro county classification. We processed each state as described above but altered the ancillary raster so that the four developed NLCD classes within metro counties were given different values than the developed classes within nonmetro counties. This analysis did not show a significant difference in RMSE when compared to our state-by-state analysis. There may be a better scheme to highlight the differing population dynamics between rural and urban areas, but it would likely require more refined data than the county level. Dmowska and Stepinski (2017) used nationally determined densities but achieved lower error by taking advantage of a national land use map (Theobald, 2014) to identify uninhabited areas. We calculated a measure of error following the methods described in Dmowska and Stepinski (2017) and our error was comparable, but higher (43.17 mean block group RMSE versus 45.21 mean block group RMSE). However, the development of the Theobald land use map required a significant effort, and it is not clear whether those data will be available beyond 2010. The methods described here resulted in a comparable product using a variety of readily available and frequently updated data sources that can be appended as new sources become available or replaced entirely when more refined locally available data identifying uninhabited areas are available. The combination of identifying uninhabited areas along with the use of local or regional zones reduced RMSE and resulted in a more accurate dasymetric population product.

The representative population densities determined from IDM (Table 2) intuitively make sense. The four developed land cover classes were consistently orders of magnitude higher than all other land cover classes for all states. The densities were higher for “developed, low intensity” compared to “developed, open space” and were almost always higher for “developed, medium intensity” compared to “developed, low intensity”. The “developed, high intensity” land cover class was, however, often lower than the medium-intensity class likely due to the influence of highly developed and lightly populated industrial and commercial areas.

IDM’s accuracy seems to be dependent on the spatial distribution of the population. States with the lowest NRMSE such as Connecticut and New Jersey tend to have larger urban areas with higher population counts well distributed throughout the state. This trend is likely from these states having a higher number of homogenous blocks from across the state identified as representative blocks. Conversely, states with the highest NRMSE such as North Dakota and Wyoming tend to be characterized by small population centers surrounded by large sparsely populated lands (Fig. 5). These states tend to have fewer, less evenly distributed blocks eligible to be representative blocks. The same pattern seems to be repeated for counties. A given state’s IDM representative population densities perform better in counties with a dispersed distribution of high population throughout the county rather than a stark difference between high-population centers and surrounding sparsely populated areas. For example, some of the counties with the highest NRMSE in central and western Montana are characterized by low-population blocks throughout the county with small concentrations of higher-population blocks (Fig. 5). Furthermore, the counties in Michigan’s upper peninsula with fewer urban areas tend to have higher NRMSE than the counties in the south with more distributed urban areas (Fig. 5).

### 5.2 Uncertainty and limitations

Dasymetric modeling assumes a predictive relationship between ancillary data and a ground truth population surface, but, like any model, it only represents an approximation, with various sources of uncertainty. The core assumption is that population density is homogenous within ancillary classes, and many studies, including this one, put emphasis on refining those ancillary classes to make them more homogenous, or to allow for a degree of spatial autocorrelation in the heterogeneity of in-class density by using different estimates in different zones. As higher-resolution ancillary data become more readily available, such efforts may face diminishing returns because a smaller spatial unit of measurement may have less sub-unit heterogeneity but more proportional uncertainty with regard to the population estimates (Azar et al., 2013; Nagle et al., 2014). Reducing uncertainty, therefore, is a matter of refining the fidelity between the ancillary data and the population density surface through a combination of automated and expert-guided techniques, often iteratively.

The decision to substitute the representative population density of shrub/scrub in Connecticut with a national average illustrates the importance of reviewing the output of IDM. Indeed, we would not expect shrub/scrub to be the most densely populated land cover class within Connecticut, and the estimated density is clearly an outlier when compared to other states’ values for that same land cover class. While there are other values in our final estimates (Table 2) that may warrant additional attention, we believed this particular representative population density was so far outside the range of the other states we needed to consider an alternative value. It is imperative to review the results for logical consistency and consider modifications based on local knowledge before accepting the results.

Data for our uninhabited areas have a wide temporal range due to the varying frequency at which they are updated. For example, OSM data reflect the most recent edits made by contributors while the NLCD roads and energy development are from 2011. Although our population estimates are from the 2010 decennial census, the uninhabited areas are not restricted to 2010. There might be additional uninhabited areas since 2010. Furthermore, the rules applied to filter and refine uninhabited areas were determined for a national allocation of population. The EnviroAtlas IDM toolbox for ArcGIS Pro (https://github.com/USEPA/Dasymetric-Toolbox-ArcGISPro, last access: 15 June 2022) or open source GIS (https://github.com/USEPA/Dasymetric-Toolbox-OpenSource, last access: 15 June 2022) can be used to refine population estimates if more detailed local or regional data for uninhabited areas are available. It is important to note that the accuracy of the population estimates is dependent on the accuracy of the input data. Some sources of uncertainty are the accuracy of the NLCD classification, the census block boundaries, and the boundaries and labels of various OSM, PAD-US, and NAVSTREETS layers.

## 6 Code and data availability

The Dasymetric Toolbox for ArcGIS Pro (https://github.com/USEPA/Dasymetric-Toolbox-ArcGISPro, last access: 15 June 2022; https://doi.org/10.5281/zenodo.6645816, US Environmental Protection Agency, 2022a) and Dasymetric Toolbox for Open Source GIS (https://github.com/USEPA/Dasymetric-Toolbox-OpenSource, last access: 15 June 2022; https://doi.org/10.5281/zenodo.6645824, US Environmental Protection Agency, 2022b) are available on the US EPA’s GitHub page. The updated EnviroAtlas dasymetric population map at 30 m resolution for the CONUS is available via the EPA’s Environmental Dataset Gateway (https://doi.org/10.23719/1522948, Baynes et al., 2021). Data can also be accessed or viewed from the EPA’s EnviroAtlas (https://www.epa.gov/enviroatlas, last access: 15 June 2022; Pickard et al., 2015). Dasymetric population estimates for US states and territories outside the CONUS are in progress. Updates for all US states and territories for the 2020 US Census are planned and will be available on the EPA’s EnviroAtlas.

Maps throughout this article were created using ArcGIS® software by Esri. ArcGIS™ and ArcMap™ are the intellectual property of Esri and are used herein under license. Copyright © Esri. All rights reserved. For more information about Esri® software, please visit https://www.esri.com (last access: 15 June 2022). Use of OpenStreetMap data requires the following acknowledgement: “Map data copyrighted OpenStreetMap contributors and available from https://www.openstreetmap.org”(last access: 15 June 2022).

## 7 Conclusions

In this study, we updated the existing dasymetric population map by the EPA’s EnviroAtlas by using additional geospatial datasets to expand the coverage of uninhabited areas. We used IDM developed by Mennis and Hultgren (2006) to estimate gridded 30 m population density for the CONUS. The improved identification and masking of uninhabited areas improved the accuracy of population estimates for all CONUS states. Our accuracy assessment method showed that the IDM method was better at mirroring the census block population counts of states with larger urban areas and smaller areas of sparsely populated land. The datasets and methods described here will be used to update the dasymetric population estimates for the CONUS once 2020 land cover and census data are available. Furthermore, the updated IDM toolbox will be used to specify uninhabited areas and to produce gridded population estimates for Alaska, Hawaii, Puerto Rico, and the Virgin Islands. The dasymetric population map and the IDM toolbox will be available in EnviroAtlas.

## Figures and Tables

**Table 1.** Datasets used for updating the EnviroAtlas dasymetric population map. IDM uses in bold were used in the 2016 EnviroAtlas dasymetric population map (m/u: possible mixed-use feature).

Source	Dataset/version/ format/data type	Data name	IDM use
US Census Bureau	Census blocks Vintage, 2010 TIGER/Line ESRI Shapefile Vector – polygon	Census blocks witd population and housing counts	Source units
Multi-Resolution Land Characteristics Consortium/National Land Cover Database	2011 Land Cover Version 2 (2016) ERDAS Imagine Raster – 30m	Developed, open space	**Ancillary class**
		Developed, low intensity	**Ancillary class**
		Developed, medium intensity	**Ancillary class**
		Developed, high intensity	**Ancillary class**
		Barren land (rock/sand/clay)	**Ancillary class**
		Evergreen forest	**Ancillary class**
		Mixed Forest	**Ancillary class**
		Shrub/scrub	**Ancillary class**
		grassland/herbaceous	**Ancillary class**
		Pasture/hay	**Ancillary class**
		Cultivated crops	**Ancillary class**
		Woody wetlands	**Ancillary class**
		Emergent herbaceous wetlands	**Ancillary class**
		Perennial ice/snow	**Ancillary class**
		Open water	**Ancillary class**
	Developed Imperviousness Descriptor 2016 Edition, 2011 ERDAS Imagine Raster – 30 m	Primary road in urban area	Uninhabited area
		Primary road outside urban area	Uninhabited area
		Energy production site in urban area	Uninhabited area
		Energy production site outside urban area	Uninhabited area
HERE/NAVSTREETS	Land Use A 9, 0, 2017 ESRI Geodatabase Vector – polygon	Shopping center	Uninhabited feature (m/u)
		Industrial complex	Uninhabited feature (m/u)
		Cemetery	Uninhabited feature
	Land Use B 9, 0, 2017 ESRI Geodatabase Vector – polygon	Aircraft roads	Uninhabited feature
OpenStreetMap Foundation (OSMF) & Contributors	Land use 2019 ESRI Shapefile Vector – polygon	Retail	Uninhabited feature (m/u)
		Commercial	Uninhabited feature (m/u)
		Mall	Uninhabited feature (m/u)
		Industrial	Uninhabited feature (m/u)
	Places of interest 2019 ESRI Shapefile Vector – line ESRI Shapefile Vector – polygon	Supermarket	Uninhabited feature (m/u)
		School	Uninhabited feature
North American Rail Network	Rail network 2019 ESRI Shapefile Vector – line	Rail network	Uninhabited feature
CoreLogic	Residential parcels 2018 ESRI Shapefile Vector – polygon	Residential parcels	Inhabited feature
US Geological Survey Gap Analysis Project/ Protected Areas Database of the US	Combined protected areas Proclamation, marine, fee designation, easement 2.0, 2018 ESRI Geodatabase Vector – polygon	Local park	Uninhabited feature
		State park	Uninhabited feature
		State forest	Uninhabited feature
		National wildlife refuge	Uninhabited feature
		National forest	Uninhabited feature
		National park	Uninhabited feature
		National lakeshore/seashore	Uninhabited feature
		National grassland	Uninhabited feature
US Geological Survey	2012 Raster – 30 m (projected to match NLCD)	National elevation dataset	**> 25 % slope = uninhabited area**

**Table 2.** Representative population densities determined using IDM. Note the heat map is scaled light blue to dark blue based on the sorted rank of densities for each state.

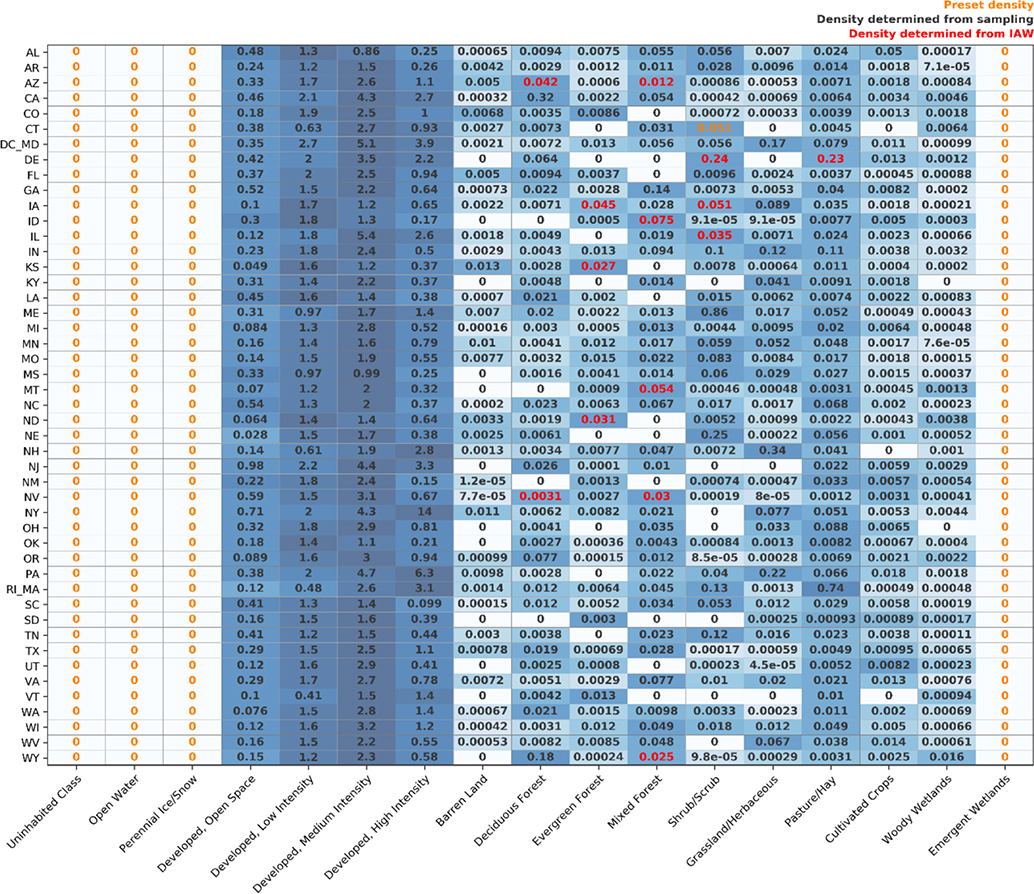

**Table 3.** Census block average error by state after applying block level representative population densities for each ancillary class to census tracts and ensuring pycnophylactic integrity at the tract level. Changes in error from the 2016 specification of uninhabited areas are in parentheses.

State	RMSE		NRMSE		MAE	

AL	36.32	(−1.48)	1.86	(−0.08)	13.13	(−0.6)
AR	33.21	(−0.92)	2.07	(−0.06)	11.50	(−0.35)
AZ	52.18	(−2.88)	1.94	(−0.11)	15.84	(−1.02)
CA	78.32	(−5.05)	1.47	(−0.09)	28.31	(−2.67)
CO	44.50	(−2.84)	1.74	(−0.11)	15.46	(−1.29)
CT	65.43	(−2.56)	1.21	(−0.05)	28.29	(−1.71)
DC_MD	75.54	(−3.13)	1.73	(−0.07)	27.81	(−1.71)
DE	64.97	(−4.45)	1.71	(−0.12)	25.09	(−1.97)
FL	66.61	(−3.29)	1.68	(−0.08)	23.05	(−1.33)
GA	59.00	(−4.44)	1.75	(−0.13)	20.58	(−1.65)
IA	28.17	(−0.99)	1.97	(−0.07)	10.25	(−0.45)
ID	23.80	(−1.46)	2.23	(−0.14)	7.51	(−0.46)
IL	49.90	(−3.37)	1.72	(−0.12)	18.92	(−1.55)
IN	41.92	(−1.74)	1.69	(−0.07)	15.62	(−0.86)
KS	25.74	(−0.99)	2.11	(−0.08)	8.38	(−0.34)
KY	43.22	(−3.17)	1.57	(−0.12)	16.54	(−1.17)
LA	44.59	(−1.22)	1.96	(−0.05)	15.59	(−0.58)
ME	36.80	(−1.56)	1.88	(−0.08)	13.10	(−0.48)
MI	41.38	(−2.47)	1.36	(−0.08)	16.14	(−1.29)
MN	36.80	(−1.87)	1.77	(−0.09)	13.05	(−0.83)
MO	32.32	(−1.28)	1.80	(−0.07)	11.26	(−0.63)
MS	39.11	(−0.98)	2.21	(−0.06)	13.08	(−0.58)
MT	19.66	(−1.68)	2.60	(−0.22)	6.08	(−0.49)
NC	48.84	(−1.99)	1.46	(−0.06)	19.83	(−0.91)
ND	17.27	(−0.76)	3.39	(−0.15)	4.61	(−0.23)
NE	21.04	(−1.41)	2.18	(−0.15)	6.91	(−0.42)
NH	46.90	(−1.81)	1.69	(−0.07)	17.33	(−0.91)
NJ	73.17	(−7.85)	1.38	(−0.15)	29.52	(−3.72)
NM	29.95	(−1.96)	2.41	(−0.16)	8.81	(−0.65)
NV	64.16	(−2.33)	1.98	(−0.07)	18.42	(−1.07)
NY	89.75	(−5.56)	1.60	(−0.1)	32.59	(−2.89)
OH	48.09	(−2.6)	1.48	(−0.08)	17.89	(−1.42)
OK	30.35	(−1.29)	2.12	(−0.09)	9.75	(−0.49)
OR	36.90	(−2.57)	1.87	(−0.13)	11.75	(−0.88)
PA	54.89	(−3.99)	1.79	(−0.13)	20.45	(−1.67)
RI_MA	63.00	(−2.18)	1.46	(−0.05)	25.41	(−1.34)
SC	44.46	(−1.93)	1.71	(−0.07)	16.53	(−0.81)
SD	23.18	(−1.77)	2.49	(−0.19)	7.52	(−0.55)
TN	44.51	(−1.99)	1.65	(−0.07)	16.63	(−0.81)
TX	58.50	(−2.41)	2.08	(−0.09)	18.47	(−1.12)
UT	41.00	(−3.08)	1.68	(−0.13)	13.38	(−1.27)
VA	63.94	(−1.3)	2.24	(−0.05)	17.45	(−1.44)
VT	34.48	(−3.03)	1.74	(−0.15)	11.83	(−1.04)
WA	51.45	(−3.72)	1.47	(−0.11)	18.99	(−1.56)
WI	36.56	(−3.27)	1.59	(−0.14)	13.61	(−1.53)
WV	27.07	(−1.49)	1.91	(−0.11)	9.58	(−0.61)
WY	19.35	(−1.47)	2.91	(−0.22)	5.79	(−0.46)

**Table 4.** Count of pixels with and without population using the 2016 specification of uninhabited and the updated specification of uninhabited. Note that counts include pixels within zero population blocks.

	Pixels with population = 0	Pixels with population > 0	Population in updated uninhabited

2016 dasymetric map	4 338 376 834	4 641 477 058	9 564 807
Updated dasymetric map	4525 111 385	4 454 742 507	-
Difference	(186 734 551)	186 734 551	9 564 807

**Table 5.** Census block error for CONUS after applying block-level representative population density for each ancillary class to census tracts and ensuring pycnophylactic integrity at the tract level.

	RMSE	NRMSE	MAE

2016 uninhabited areas (nationally determined densities)	58.14	2.04	4.48
Updated uninhabited areas (nationally determined densities)	55.31	1.95	4.34
2016 uninhabited areas (state-by-state determined densities)	54.80	1.93	4.30
Updated uninhabited areas (state-by-state determined densities)	51.93	1.83	4.15

## References

[R1] Azar D, Engstrom R, Graesser J, and Comenetz J: Generation of fine-scale population layers using multi-resolution satellite imagery and geospatial data, Remote Sens. Environ., 130, 219–232, 10.1016/j.rse.2012.11.022, 2013.

[R2] Baynes J, Neale A, and Hultgren T: 2010 Dasymetric Population for the Conterminous United States v3, US Environmental Protection Agency Office of Research and Development [data set], 10.23719/1522948, 2021.

[R3] Bellwood DR, Hoey AS, and Hughes TP: Human activity selectively impacts the ecosystem roles of parrot-fishes on coral reefs, Proc. Biol. Sci, 279, 1621–1629, 10.1098/rspb.2011.1906, 2012. 22090383 PMC3282342

[R4] Carroll RJ, Chen R, George EI, Li TH, Newton HJ, Schmiediche H, and Wang N: Ozone Exposure and Population Density in Harris County, Texas, J. Am. Stat. Assoc, 92, 392–404, 10.1080/01621459.1997.10473988, 1997.

[R5] Cinner JE, Graham NA, Huchery C, and Macneil MA: Global effects of local human population density and distance to markets on the condition of coral reef fisheries, Conserv. Biol, 27, 453–458, 10.1111/j.1523-1739.2012.01933.x, 2013. 23025334

[R6] CoreLogic: CoreLogic Parcel, HIFLD [data set], https://hifld-geoplatform.hub.arcgis.com (last access: 15 June 2022), 2018.

[R7] Dmowska A and Stepinski TF: A high resolution population grid for the conterminous United States: The 2010 edition, Comput. Environ. Urban, 61, 13–23, 10.1016/j.compenvurbsys.2016.08.006, 2017.

[R8] Dobson JE, Bright EA, Coleman PR, Durfee RC, and Worley BA: LandScan: A Global Population Database for Estimating Populations at Risk, Photogramm. Eng. Rem. S., 66, 7, 849–857, 2000.

[R9] Fang Y and Jawitz JW: High-resolution reconstruction of the United States human population distribution, 1790 to 2010, Sci. Data, 5, 180067, 10.1038/sdata.2018.67, 2018. 29688219 PMC5914287

[R10] Fang Y and Jawitz JW: The evolution of human population distance to water in the USA from 1790 to 2010, Nat. Commun, 10, 430, 10.1038/s41467-019-08366-z, 2019. 30683855 PMC6347611

[R11] Fang Y, Ceola S, Paik K, McGrath G, Rao PSC, Montanari A, and Jawitz JW: Globally Universal Fractal Pattern of Human Settlements in River Networks, Earth’s Future, 6, 1134–1145, 10.1029/2017ef000746, 2018.

[R12] Federal Railroad Administration: North American Rail Lines, Bureau of Transportation Statistics [data set], https://www.bts.gov/maps (last access: 15 June 2022), 2019.

[R13] GDAL/OGR contributors: GDAL/OGR Geospatial Data Abstraction Software Library, Open Source Geospatial Foundation [code], https://gdal.org (last access: 15 June 2022), 2019.

[R14] Gergely KJ and McKerrow A: PAD-US – National inventory of protected areas (ver. 1.1, August 2016): US Geological Survey Fact Sheet 2013–3086, Report, 2, 10.3133/fs20133086, 2016.

[R15] Goodchild MF and Lam NS-N: Areal interpolation: A variant of the traditional spatial problem, Geo-Processing, 1, 297–312, 1980.

[R16] HERE: NAVSTREETS Streets Data, HERE [data set], https://www.here.com/(last access: 15 June 2022), 2017.

[R17] Homer C, Dewitz J, Jin S, Xian G, Costello C, Danielson P, Gass L, Funk M, Wickham J, Stehman S, Auch R, and Riitters K: Conterminous United States land cover change patterns 2001–2016 from the 2016 National Land Cover Database, ISPRS J. Photogramm., 162, 184–199, 10.1016/j.isprsjprs.2020.02.019, 2020. PMC9214659 35746921

[R18] Jones KE, Patel NG, Levy MA, Storeygard A, Balk D, Gittleman JL, and Daszak P: Global trends in emerging infectious diseases, Nature, 451, 990–993, 10.1038/nature06536, 2008. 18288193 PMC5960580

[R19] Karunarathne A and Lee G: Estimating Hilly Areas Population Using a Dasymetric Mapping Approach: A Case of Sri Lanka’s Highest Mountain Range, ISPRS Int. Geo-Inf., 8, 166, 10.3390/ijgi8040166, 2019.

[R20] Leyk S, Gaughan AE, Adamo SB, de Sherbinin A, Balk D, Freire S, Rose A, Stevens FR, Blankespoor B, Frye C, Comenetz J, Sorichetta A, MacManus K, Pistolesi L, Levy M, Tatem AJ, and Pesaresi M: The spatial allocation of population: a review of large-scale gridded population data products and their fitness for use, Earth Syst. Sci. Data, 11, 1385–1409, 10.5194/essd-11-1385-2019, 2019.

[R21] Liu C, Wang F, and Xu Y: Habitation environment suitability and population density patterns in China: A regionalization approach, Growth Change, 50, 184–200, 10.1111/grow.12283, 2019.

[R22] Lloyd CT, Chamberlain H, Kerr D, Yetman G, Pistolesi L, Stevens FR, Gaughan AE, Nieves JJ, Hornby G, MacManus K, Sinha P, Bondarenko M, Sorichetta A, and Tatem AJ: Global spatio-temporally harmonised datasets for producing high-resolution gridded population distribution datasets, Big Earth Data, 3, 108–139, 10.1080/20964471.2019.1625151, 2019. 31565697 PMC6743742

[R23] Mennis J and Hultgren T: Intelligent Dasymetric Mapping and Its Application to Areal Interpolation, Cartogr. Geogr. Inf. Sc., 33, 179–194, 10.1559/152304006779077309, 2006.

[R24] Moos M, Vinodrai T, Revington N, and Seasons M: Planning for Mixed Use: Affordable for Whom?, J. Am. Plann. Assoc., 84, 7–20, 10.1080/01944363.2017.1406315, 2018.

[R25] Morais RA, Connolly SR, and Bellwood DR: Human exploitation shapes productivity-biomass relationships on coral reefs, Glob. Chang. Biol, 26, 1295–1305, 10.1111/gcb.14941, 2019. 31782858

[R26] Nagle NN, Buttenfield BP, Leyk S, and Spielman S: Dasymetric Modeling and Uncertainty, Ann. Assoc. Am. Geogr., 104, 80–95, 10.1080/00045608.2013.843439, 2014. 25067846 PMC4109831

[R27] Nahayo L, Ndayisaba F, Karamage F, Nsengiyumva JB, Kalisa E, Mind’je R, Mupenzi C, and Li L: Estimating landslides vulnerability in Rwanda using analytic hierarchy process and geographic information system, Integr. Environ. Assess. Manag, 15, 364–373, 10.1002/ieam.4132, 2019. 30702199

[R28] Nasiri H, Yusof MJM, Ali TAM, and Hussein MKB: District flood vulnerability index: urban decision-making tool, Int. J. Environ. Sci. Te., 16, 2249–2258, 10.1007/s13762-018-1797-5, 2018.

[R29] Nicholls RJ and Small C: Improved Estimates of Coastal Population and Exposure to Hazards Released, EOS, 83, 301–305, 2002.

[R30] OpenStreetMap contributors: Planet dump, Planet OSM [data set], https://planet.osm.org (last access: 15 June 2022), 2019.

[R31] Pickard BR, Daniel J, Mehaffey M, Jackson LE, and Neale A: EnviroAtlas: A new geospatial tool to foster ecosystem services science and resource management, Ecosyst. Serv., 14, 45–55, 10.1016/j.ecoser.2015.04.005, 2015.

[R32] Radeloff VC, Stewart SI, Hawbaker TJ, Gimmi U, Pidgeon AM, Flather CH, Hammer RB, and Helmers DP: Housing growth in and near United States protected areas limits their conservation value, Proc. Natl. Acad. Sci. USA, 107, 940–945, 10.1073/pnas.0911131107, 2010. 20080780 PMC2818924

[R33] Samoli E, Stergiopoulou A, Santana P, Rodopoulou S, Mitsakou C, Dimitroulopoulou C, Bauwelinck M, de Hoogh K, Costa C, Mari-Dell’Olmo M, Corman D, Vardoulakis S, Katsouyanni K, and Consortium E-H: Spatial variability in air pollution exposure in relation to socioeconomic indicators in nine European metropolitan areas: A study on environmental inequality, Environ. Pollut, 249, 345–353, 10.1016/j.envpol.2019.03.050, 2019. 30909127

[R34] Samson J, Berteaux D, McGill BJ, and Humphries MM: Geographic disparities and moral hazards in the predicted impacts of climate change on human populations, Global Ecol. Biogeogr, 20, 532–544, 10.1111/j.1466-8238.2010.00632.x, 2011.

[R35] Schmidt WP, Suzuki M, Thiem VD, White RG, Tsuzuki A, Yoshida LM, Yanai H, Haque U, Tho le H, Anh DD, and Ariyoshi K: Population density, water supply, and the risk of dengue fever in Vietnam: cohort study and spatial analysis, PLoS Med, 8, e1001082, 10.1371/journal.pmed.1001082, 2011. 21918642 PMC3168879

[R36] Smith A, Bates PD, Wing O, Sampson C, Quinn N, and Neal J: New estimates of flood exposure in developing countries using high-resolution population data, Nat. Commun, 10, 1814, 10.1038/s41467-019-09282-y, 2019. 31000721 PMC6472407

[R37] Song Y and Knaap G-J: Measuring Urban Form: Is Portland Winning the War on Sprawl?, J. Am. Plann. Assoc., 70, 210–225, 2004.

[R38] Sorichetta A, Hornby GM, Stevens FR, Gaughan AE, Linard C, and Tatem AJ: High-resolution gridded population datasets for Latin America and the Caribbean in 2010, 2015, and 2020, Sci. Data, 2, 150045, 10.1038/sdata.2015.45, 2015. 26347245 PMC4555876

[R39] Stevens FR, Gaughan AE, Linard C, and Tatem AJ: Disaggregating census data for population mapping using random forests with remotely-sensed and ancillary data, PLoS One, 10, e0107042, 10.1371/journal.pone.0107042, 2015. 25689585 PMC4331277

[R40] Taubenböck H, Weigand M, Esch T, Staab J, Wurm M, Mast J, and Dech S: A new ranking of the world’s largest cities – Do administrative units obscure morphological realities?, Remote Sens. Environ, 232, 111353, 10.1016/j.rse.2019.111353, 2019.

[R41] Theobald DM: Development and Applications of a Comprehensive Land Use Classification and Map for the US, PLOS ONE, 9, e94628, 10.1371/journal.pone.0094628, 2014. 24728210 PMC3984247

[R42] Tobler WR: Smooth Pycnophylactic Interpolation for Geographical Regions, J. Am. Stat. Assoc, 74, 519–530, 10.1080/01621459.1979.10481647, 1979. 12310706

[R43] US Census Bureau: Special Release – Census Blocks with Population and Housing Counts, TIGER/Line Shapefiles [data set], https://www.census.gov/geographies/mapping-files/2010/geo/tiger-line-file.html (last access: 15 June 2022), 2012.

[R44] US Census Bureau: 2010 TIGER/Line Shapefiles Technical Documentation, https://www2.census.gov/geo/pdfs/maps-data/data/tiger/tgrshp2010/TGRSHP10SF1.pdf (last access: 15 June 2022), 2012.

[R45] US Department of Agriculture (Economic Research Service): 2013 Rural-Urban Continuum Codes, USDA [data set], https://www.ers.usda.gov/data-products/rural-urban-continuum-codes.aspx (last access: 15 June 2022), 2020.

[R46] US Environmental Protection Agency: The Benefits and Costs of the Clean Air Act from 1990 to 2020, Final Report – Rev. A, https://www.epa.gov/sites/default/files/2015-07/documents/fullreport_rev_a.pdf (last access: 15 June 2022), 2011.

[R47] US Environmental Protection Agency: USEPA/Dasymetric-Toolbox-ArcGISPro: v1.0.0 (v1.0.0), Zenodo [code], 10.5281/zenodo.6645816, 2022a.

[R48] US Environmental Protection Agency: USEPA/Dasymetric-Toolbox-OpenSource: v1.0.0 (v1.0.0), Zenodo [code], 10.5281/zenodo.6645824, 2022b.

[R49] US Geological Survey, EROS Data Center: USGS 30 Meter Resolution, One-Sixtieth Degree National Elevation Dataset for CONUS, Alaska, Hawaii, Puerto Rico, and the U. S. Virgin Islands, US Geological Survey [data set], https://www.usgs.gov/programs/national-geospatial-program/national-map (last access: 15 June 2022), 1999.

[R50] US Geological Survey, Gap Analysis Program: Protected Areas Database of the United States (PAD-US), US Geological Survey [data set], 10.5066/P955KPLE, 2018.

[R51] Venter O, Sanderson EW, Magrach A, Allan JR, Beher J, Jones KR, Possingham HP, Laurance WF, Wood P, Fekete BM, Levy MA, and Watson JE: Sixteen years of change in the global terrestrial human footprint and implications for biodiversity conservation, Nat. Commun, 7, 12558, 10.1038/ncomms12558, 2016. 27552116 PMC4996975

[R52] Weber EM, Seaman VY, Stewart RN, Bird TJ, Tatem AJ, McKee JJ, Bhaduri BL, Moehl JJ, and Reith AE: Census-independent population mapping in northern Nigeria, Remote Sens. Environ, 204, 786–798, 10.1016/j.rse.2017.09.024, 2018. 29302127 PMC5738969

[R53] Wei C, Taubenböck H, and Blaschke T: Measuring urban agglomeration using a city-scale dasymetric population map: A study in the Pearl River Delta, China, Habitat Int, 59, 32–43, 10.1016/j.habitatint.2016.11.007, 2017.

[R54] Wickham J, Stehman SV, Sorenson DG, Gass L, and Dewitz JA: Thematic accuracy assessment of the NLCD 2016 land cover for the conterminous United States, Remote Sens. Environ, 257, 112357, 10.1016/j.rse.2021.112357, 2021. PMC6657805 31346298

[R55] Yang L, Jin S, Danielson P, Homer C, Gass L, Bender SM, Case A, Costello C, Dewitz J, Fry J, Funk M, Granneman B, Liknes GC, Rigge M, and Xian G: A new generation of the United States National Land Cover Database: Requirements, research priorities, design, and implementation strategies, ISPRS J. Photogramm, 146, 108–123, 10.1016/j.isprsjprs.2018.09.006, 2018.

[R56] Ye T, Zhao N, Yang X, Ouyang Z, Liu X, Chen Q, Hu K, Yue W, Qi J, Li Z, and Jia P: Improved population mapping for China using remotely sensed and points-of-interest data within a random forests model, Sci. Total Environ, 658, 936–946, 10.1016/j.scitotenv.2018.12.276, 2019. 30583188

[R57] Yuan H, Gao X, and Qi W: Fine-Scale Spatiotemporal Analysis of Population Vulnerability to Earthquake Disasters: Theoretical Models and Application to Cities, Sustainability, 11, 2149, 10.3390/su11072149, 2019.

